# *Cryptosporidium *infection in a veal calf cohort in France: molecular characterization of species in a longitudinal study

**DOI:** 10.1186/1297-9716-42-116

**Published:** 2011-12-02

**Authors:** Jérôme Follet, Karine Guyot, Hélène Leruste, Anne Follet-Dumoulin, Ourida Hammouma-Ghelboun, Gabriela Certad, Eduardo Dei-Cas, Patrice Halama

**Affiliations:** 1 Laboratoire Biotechnologies et Gestion des Agents Pathogènes, Institut Supérieur d'Agriculture, 59048 Lille, France; 2 Laboratoire Biologie et Diversité des Pathogènes Eucaryotes Emergents, Institut Pasteur de Lille, 59019 Lille, France; 3 INSERM U1019, Institut Pasteur de Lille, 59019 Lille, France; 4 CNRS UMR8204, Institut Pasteur de Lille, 59019 Lille, France; 5 Laboratoire Environnement et Santé, Faculté Libre des Sciences et Technologies, 59048 Lille, France

## Abstract

Feces from 142 animals were collected on 15 farms in the region of Brittany, France. Each sample was directly collected from the rectum of the animal and identified with the ear tag number. Animals were sampled three times, at 5, 15 and 22 weeks of age. After DNA extraction from stool samples, nested PCR was performed to amplify partial 18S-rDNA and 60 kDa glycoprotein genes of *Cryptosporidium*. The parasite was detected on all farms. One hundred out of 142 calves (70.4%) were found to be parasitized by *Cryptosporidium*. Amplified fragments were sequenced for *Cryptosporidium *species identification and revealed the presence of *C. parvum *(43.8%)*, C. ryanae *(28.5%), and *C. bovis *(27%). One animal was infected with *Cryptosporidium ubiquitum*. The prevalence of these species was related to the age of the animal. *C. parvum *caused 86.7% of *Cryptosporidium *infections in 5-week-old calves but only 1.7% in 15-week-old animals. The analysis of the results showed that animals could be infected successively by *C. parvum*, *C. ryanae*, and *C. bovis *for the study period. *C. parvum *gp60 genotyping identifies 6 IIa subtypes of which 74.5% were represented by IIaA15G2R1. This work confirms previous studies in other countries showing that zoonotic *C. parvum *is the dominant species seen in young calves.

## Introduction

*Cryptosporidium *is a genus of protozoan parasites infecting a wide range of hosts [1]. All groups of vertebrates are susceptible to *Cryptosporidium *infection worldwide. This parasite is the etiological agent of cryptosporidiosis, which is mainly characterized by diarrhea in humans and livestock. Massive outbreaks of enteritis in people such as in Milwaukee, Wisconsin (USA) have increased public awareness of this parasite [2]. In humans, the prevalence and severity of infection increase in immunodeficient individuals such as AIDS patients. In immunocompetent patients, the disease is self-limited [3]. No drug therapy is yet available and the high resistance of oocysts to environmental conditions and chemical treatment make cryptosporidiosis difficult to control [4]. Cattle have been considered to be a primary reservoir for *Cryptosporidium *oocysts for zoonotic *C. parvum *[5]. These animals could be a risk factor via environmental contamination from their manure being spread on farmland or their grazing on watersheds [6]. On farms, transmission of *Cryptosporidium *spp. can result from ingestion of contaminated food or water, by direct transmission from host to host, or through insect vectors [7]. In cattle, infection by *Cryptosporidium *spp. was first reported in 1971 [8]. Since vaccines have become commercially available against *Escherichia coli *K99, rotavirus, and coronavirus, *Cryptosporidium *has emerged as the main neonatal diarrheic agent in calves [9]. In farm animals, the economic impact is related to morbidity, mortality and growth retardation [10]. Among the 24 species previously described (if the three fish species are accepted without complete genetic characterization) [1,11-13], *C. parvum, C. bovis, C. ryanae *and *C. andersoni *usually infect cattle. *C. parvum *has zoonotic potential and is a frequent cause of human cryptosporidiosis [14]. *C. bovis *and *C. ryanae *have not been found in humans and there is only one description of *C. andersoni *in a patient [15]. Recent reports have described an age-related distribution of these aforementioned species in dairy cattle on the east coast of the United States [16-18], India, China, Georgia [19], Malaysia [20], and Denmark [21]. The most prevalent species were *C. parvum *in preweaned calves, *C. ryanae *and *C. bovis *in postweaned calves and *C. andersoni *in adult cows [16,17].

In France, previous studies on the prevalence of *Cryptosporidium *in cattle were based on microscopic determination [22] or coproantigen detection using ELISA [23]. These studies on dairy calves reported a within herd prevalence of *Cryptosporidium *without identifying species or the relation to the host's age. The present study was conducted in 15 farms in Brittany, France to determine the prevalence of *Cryptosporidium *in veal calves. We used genotyping and subtyping for the molecular study of *Cryptosporidium *isolates. Follow-up of the same animal allowed us to determine the outcome of the infection and the age distribution of *Cryptosporidium *species.

## Material and methods

### Specimen sources and collection

**Figure 1:**
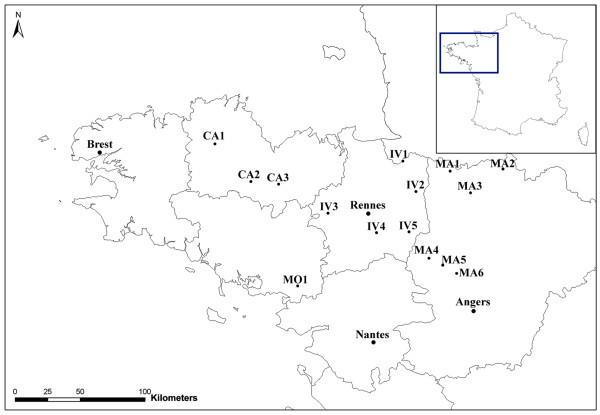
**Map of administrative regions in Brittany showing the location of farms included in the study: Côtes d'Armor (CA), Ile-et-Vilaine (IV), Mayenne (MA), and Morbihan (MO) in France**.

Fifteen fattening units in Brittany (France) were included in this work. They belonged to three industrial veal producers representative of integrators in France and did not present any known history of *Cryptosporidium *infection. These farms were located in four administrative regions (Figure 1): Côtes d'Armor (CA1-CA3), Morbihan (MO1), Ile-et-Vilaine (IV1-IV5), and Mayenne (MA1-MA6). During the summer and autumn of 2007, all farms were visited three times and fecal samples were taken from 142 animals exhibiting diarrhea at the age of 5 weeks old. Calves arrived in fattening units at the age of 2 weeks old and were confined in small groups from four to six animals per pen. Because of a concomitant welfare study [24], calves had to stay 2 to 3 weeks without any external stress despite the farmer's presence. At the age of 22 weeks old, calves were finally sent to the slaughterhouse. Consequently, sampling was done at the ages of 5 weeks, 15 weeks, and 22 weeks (Table 1). These points of sampling corresponded to the beginning, the middle and the end of the fattening period. Fecal samples were collected and shipped by a veterinarian. Collectors respected the following criteria: use of a single pair of latex gloves per animal, a single plastic sterile cup per animal, and collection of at least 5 g of feces per sample. Feces were collected directly from the rectum of each animal and stored at 4 °C in potassium dichromate (2.5% wt/vol) until processed. Cups were capped, labeled with the animal's ear tag number, and accompanied by a form recording the date of sampling, the animal's sex, breed, identification number, and the mean age of the herd.

**Table 1:** *Cryptosporidium *prevalence in veal herds found in Brittany farms according to animal age.

	Animal age			
		
Farm	5 weeks No. positive/No. sample (%)	15 weeks No. positive/No. sample (%)	22 weeks No. positive/No. sample (%)	Total number of positive animals* (%)
CA1	1/6 (16.6%)	2/6 (33.3%)	1/6 (16.6%)	4/6 (66.6%)
CA2	3/10 (30%)	4/10 (40%)	3/10 (30%)	8/10 (80%)
CA3	4/10 (40%)	6/10 (60%)	0/10 (0%)	6/10 (60%)
MO1	0/10 (0%)	0/10 (0%)	1/10 (10%)	1/10 (10%)
IV1	6/10 (60%)	7/10 (70%)	1/10 (10%)	10/10 (100%)
IV2	4/10 (40%)	4/10 (40%)	3/10 (30%)	6/10 (60%)
IV3	2/8 (25%)	7/8 (87.5%)	3/8 (37.5%)	8/8 (100%)
IV4	3/10 (30%)	4/10 (40%)	0/10 (0%)	6/10 (60%)
IV5	4/10 (40%)	2/10 (20%)	3/10 (30%)	5/10 (50%)
MA1	8/10 (80%)	3/10 (30%)	1/10 (10%)	9/10 (90%)
MA2	7/10 (70%)	3/10 (30%)	1/10 (10%)	7/10 (70%)
MA3	6/10 (60%)	6/9** (66.6%)	0/9** (0%)	8/10 (80%)
MA4	6/10 (60%)	4/9** (44.4%)	1/9** (11.1%)	6/10 (60%)
MA5	7/8 (87.5%)	6/8 (75%)	0/8 (0%)	8/8 (100%)
MA6	7/10 (70%)	3/10 (30%)	2/10 (20%)	8/10 (80%)
Total	68/142 (47.9%)	59/140 (42.1%)	20/140 (14.3%)	100/142 (70.4%)

* A calf is considered to be positive if at least one out of the three samples is positive.

**The number of animals is 9 because one calf died between the age of 5 and 15 weeks.

### *Cryptosporidium *detection

After washing steps in water to eliminate potassium dichromate from the samples, DNA was extracted according to the method previously described [25] without the Cetyl TrimethylAmmonium Bromide (CTAB) and PolyVinylPyrrolidone (PVP) treatment steps. An 18S RNA gene fragment was amplified by nested PCR according to Xiao *et al*. [26]. The partial gp60 gene was amplified according to Gatei et al., [27]. PCR products were analyzed on 2% agarose gel and visualized by ethidium bromide staining. To ensure purity and limit the presence of PCR inhibitors, all PCR-negative samples were reprocessed. Samples were treated for oocyst purification by immunomagnetic separation (Dynabeads ^®^anti-*Cryptosporidium*, Invitrogen ™, Norway) according to the manufacturer's instructions. These samples were finally processed as previously for DNA extraction and PCR amplification.

### *Cryptosporidium *species identification

PCR products were purified on an ultracel YM50 membrane (Microcon, Millipore, Bedford, MA, USA) according to the manufacturer's instructions. DNA sequencing reactions were performed using internal primers of the nested PCR with the ABI Prism Big Dye Terminator cycle sequencing kit (Applied Biosystem, Foster City, CA, USA). Capillary electrophoresis was performed by Genoscreen (Lille, France). Sequences were analyzed using BLAST at NCBI [28].

## Results

### *Cryptosporidium *prevalence

The prevalence of *Cryptosporidium *infection on 15 farms from four administrative regions in Brittany (France) was studied (Figure 1). All *Cryptosporidium*-positive specimens generated the expected SSU-RNA products in nested PCR and revealed that no farm was free of *Cryptosporidium*. The molecular analysis of 422 fecal samples revealed that 147 (34.8%) were positive for *Cryptosporidium*. As shown in Table 1, the overall prevalence of infected animals was 70.4% (100/142) and ranged from 10% on a farm in Morbihan (MO1) to 100% on farms in Ile-et-Vilaine (IV1, IV3) and in Mayenne (MA5). Amongst the specimens sampled from 5-week-old and 15-week-old animals, *Cryptosporidium *prevalence was 47.9% and 42.1%, respectively (range, 0%-87.5%). In 22-week-old calves, the prevalence decreased to 14.3% (range, 0%-37.5%). The prevalence of infection decreased as the age of the calves increased.

### *Cryptosporidium *species identification by 18S rDNA sequencing

For species identification, the 147 positive nested PCR products were sequenced. Sequence analysis from 137 readable electrophoregrams revealed the presence of *C. parvum*, *C. bovis*, and *C. ryanae*. One additional *Cryptosporidium *genotype showing 99% identity with *Cryptosporidium ubiquitum *(EU827413) (previously identified as *Cryptosporidium *cervine genotype [13]) was detected in one calf. This sequence was deposited in GenBank under the accession number GU124629. Sixty (43.8%) samples were identified as *C. parvum *as follows: forty-six sequences had 100% identity with the GenBank AF093490 nucleotide sequence, 11 had 100% identity with the AF308600 nucleotide sequence and three had 99% identity compared to both references. These sequences were deposited in GenBank under the accession numbers GU124615 to GU124617. For the other positive specimens, 39 (28.5%) were identified as *C. ryanae *(previously described as *Cryptosporidium *deer-like genotype). Thirty-one of these had 100% identity with the AY587166 sequence [17] and eight were 99% identical to this reference. These nucleotide sequences were deposited in GenBank under the accession numbers GU124621 to GU124628. For the last positive samples, 37 (27%) had an identical nucleotide sequence with *C. bovis *(GenBank accession number, AY120911) formerly known as the *Cryptosporidium *Bovine B genotype. Within these sequences, 34 had 100% identity to the reference deposited in GenBank, three sequences had 99% identity. These last sequences were deposited in Genbank under the accession numbers GU124618 to GU124620.

### Prevalence of *C. parvum*, *C. ryanae*, and *C. bovis *in relation to calf age

**Figure 2:**
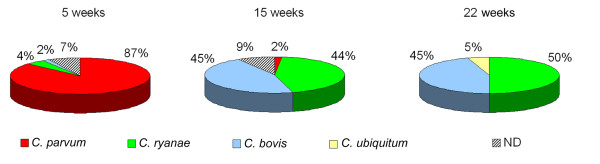
**Prevalence of *Cryptosporidium *species/genotype: *C. parvum*, *C. ryanae*, *C. bovis*, *C. C.ubiquitum *and not determined species because of unreadable sequences (ND) in calves from 5 weeks to 22 weeks of age**.

The distribution of *Cryptosporidium *species identified in animals at the age of 5, 15, and 22 weeks is shown in Figure 2. The prevalence of each species changed with the age of the calves. *C. parvum *prevalence was 86.7% in the 5-week-old calves and decreased to 1.7% in 15-week-old animals. This species was not identified in 22-week-old calves. *C. ryanae *and *C. bovis *were identified in 5-week-old calves in 4.4% and 1.5% of the specimens, respectively. The prevalence of these species in 15-week-old animals increased to 44.1% and 45.7%, respectively. This prevalence evolved to 50% and 45% in 22-week-old animals.

### Sequential infection profile

The presence of one, two, or three species of *Cryptosporidium *was determined in each animal (*n *= 91) for which the sequences were readable in all positive samples. Three calves positive for *C. parvum *at the age of 5 weeks were excluded because *Cryptosporidium *species could not be identified in all of the following samples collected in these animals. As shown in Table 2, *Cryptosporidium *species determination over time showed that only one species was identified in 63.7% (58/91) of the animals analyzed. Thus, 35.1% (32/91) had excreted only *C. parvum*, 15.4% (14/91) shed only *C. ryanae*, and 12.1% (11/91) only *C. bovis*. The *C. ubiquitum *identified in one sample accounted for 1.1%. In the time lapse of this study, 34% of the animals (31/91) were found to excrete two different species of *Cryptosporidium *successively. Indeed, 13.2% (12/91) produced *C. parvum *and *C. ryanae*, 12.1% (11/91) excreted *C. parvum *and *C. bovis*, and 8.8% (8/91) excreted *C. ryanae *and *C. bovis*. Finally, 2.2% (2/91) of the animals studied were detected to produce *C. parvum*, *C. ryanae*, and *C. bovis*.

**Table 2:** Number of *Cryptosporidium *species identified in animals and sequential infection.

**No**. *Cryptosporidium* species/genotype per animal	5 weeks	15 weeks	22 weeks	n
	*C. parvum*			31
	*C. ryanae*			2
		*C. ryanae*		10
		*C. bovis*		7
		*C. parvum*		1
			*C. bovis*	2
1			*C. ryanae*	1
			*C. ubiquitum*	1
		*C. bovis*	*C. bovis*	2
		*C. ryanae*	*C. ryanae*	1

	*C. parvum*	*C. ryanae*		10
	*C. parvum*	*C. bovis*		10
	*C. ryanae*	*C. bovis*		1
		*C. bovis*	*C. ryanae*	4
2		*C. ryanae*	*C. bovis*	2
	*C. parvum *		*C. ryanae*	1
	*C. parvum*	*C. ryanae*	*C. ryanae*	1
	*C. parvum*	*C. bovis*	*C. bovis*	1
	*C. bovis*	*C. ryanae*	*C. ryanae*	1

3	*C. parvum*	*C. ryanae*	*C. bovis*	1
	*C. parvum*	*C. bovis*	*C. ryanae*	1

ND*	*C. parvum*	ND	ND	3

ND: not determined due to unreadable sequence.

### *Cryptosporidium parvum *subtyping by gp60 sequence analysis

The subtyping analysis was performed on *C. parvum *positive specimens. From 60 targeted samples, 51 could be used for sequence analysis. As shown in Table 3, all alleles identified belong to the IIa family. The most common subtype IIaA15G2R1 (100% identity with reference strain AB 514090) was found in 38 out of 51 samples (74.51%). Six samples (11.76%) were typed as subtype IIaA17G1R1 (100% identity with reference strain GQ983359), three samples (5.89%) as subtype IIaA16G3R1 (100% identity with reference strain DQ192506) and two samples (3.92%) as subtype IIaA16G2R1 (100% identity with reference strain DQ192505). Finally one sample (1.96%) was subtyped as IIaA16G1R1 (100% identity with reference strain DQ192504) and another one (1.96%) as subtype IIaA13G1R1 (100% identity with reference strain DQ192502).

**Table 3:** gp60 gene subtypes of *C. parvum *positive samples.

Sub-genotype	No/No tot samples (%)	% identity with reference	Reference sequence in GenBank
IIaA15G2R1	38/51 (74.51%)	100	AB514090

IIaA17G1R1	6/51 (11.76%)	100	GQ983359

IIaA16G3R1	3/51 (5.89%)	100	DQ192506

IIaA16G2R1	2/51 (3.92%)	100	DQ192505

IIaA16G1R1	1/51 (1.96%)	100	DQ192504

IIaA13G1R1	1/51 (1.96%)	100	DQ192502

*Total number of samples (No tot samples) = 51 because 9 *C. parvum *positive samples gave no readable sequence for the gp60 gene marker.

## Discussion

Calves under 1 month of age are frequently infected with *Cryptosporidium sp *[29] which results in economic loss [10]. In France, up to date, the prevalence of *Cryptosporidium *in diarrheic calves has been studied only by Elisa and microscopic strategies [22,23,30]. No data are available on a molecular basis to study *Cryptosporidium *species in calf herds in that country. The present study based on 18S rDNA and gp60 gene analysis is the first in France to include molecular characterization to describe the prevalence and the host age related susceptibility to different *Cryptosporidium *species after a follow up of the same animal.

Our results showed that all fifteen farms were contaminated with *Cryptosporidium*. The parasite prevalence on farms ranged from 10% to 100% of the sampled animals. This observation was in accordance with results in Michigan (USA) where this parameter ranged from 0% to 100% [31]. The prevalence of 70.4% obtained in this work tended toward the upper end of the scale compared to other investigations done in France which ranged from 15.6% in beef herds [30] to 95% in suckling calves [23] and in other European countries where prevalence ranged from 3.4% to 96% [32,33]. However, the sampling program did not allow the study of animals under 5 weeks of age. Indeed, the animals arrived in these structures at the age of 2 to 3 weeks and farmers did not allow sampling before two complete resting weeks for each animal. Therefore, our results could underestimate the real prevalence as Huetink et al. showed that the percentage of parasite excreting animal declines after the third week of age [34] and that the first peak of prevalence is at the age of 15 days [17].

In our study, the higher prevalence of cryptosporidiosis was observed in calves 5 weeks old (47.9%) and the lowest (14.3%) in the 22-week-old animals. This observation shows that prevalence of *Cryptosporidium *infection decreases with increasing age of the cattle in France as in many other countries [17,19,33-38].

Additionally, our data confirmed the presence in France of a host age-related susceptibility to the infection with different *Cryptosporidium *species. *C. parvum *was predominantly detected in 5-week-old calves (86.7%) compared to *C. ryanae *or *C. bovis *detected in 4.4% and 1.5% of the positive samples respectively. It is noteworthy that these results are very similar to data obtained in Ireland on calves under 30 days of age with 95%, 3.6%, and 1.3% of prevalence of the same species, respectively [39] and in the UK on animals over 3 weeks old with 93% *C. parvum*, 6% *C. bovis*, and 2% *C. ryanae *[40]. In contrast to previous studies [17,41], *C. ryanae *and *C. bovis *were found with similar prevalence predominantly in 15 week and 22 week old calves. This association between the age of the cattle and the *Cryptosporidium *species identification has been supported by several studies [17,19,21,38,40] but different reports suggest that *Cryptosporidium *species repartition regarding the age of the host could be due to a technical artifact. Despite the fact that the methodological strategy based on PCR using genus specific primers and partial direct sequencing of the 18S rDNA is commonly used to identify *Cryptosporidium *species [42], this molecular tool is limited in the case of mixed infections. Feng et al., [19] suggested that the important shedding of *C. parvum *in preweaned calves had probably masked the concurrent infection of these animals by *C. bovis *or *C. ryanae*. Furthermore, previous reports suggested that a dominant *Cryptosporidium *species in a sample can be preferentially amplified by PCR [43,44]. It is noteworthy that this situation of mixed *Cryptosporidium *species infection in farm animals would be more prevalent than originally believed [45-47]. Mixed *Cryptosporidium *species could also explain sequencing difficulties encountered in this work. The simultaneous presence of several species in the same sample could lead to amplification and sequencing of different genetic fragments leading to unreadable superimposition of electrophoregrams.

Consequently, in our work based on the utilization of *Cryptosporidium *generic primers, the amplification of a single fragment with a single sequence is not conclusive evidence that the sample contains only a single species. However, based on our results, it is possible to confirm the predominance of different species of *Cryptosporidium *by group of age among the calves.

Particularly, our data showed that animals can be sequentially infected with *C. parvum, C. ryanae *and *C. bovis *as well as *C. parvum, C. bovis *and *C. ryanae*. This observation provides evidence that a previous infection with *C. parvum *did not protect calves against an infection with other *Cryptosporidium *species. Fayer et al. suggested that the peak of cryptosporidiosis prevalence in young calves could reflect the immaturity of the immune status [48]. It was also suggested that the low excretion of *C. parvum *oocysts in older calves might be related to the development of immunity that also protected the animal against a secondary challenge [49]. It has been reported that immunity arises in the first two weeks after infection [50]. Interestingly, Fayer et al. [51] described that calves previously challenged with *C. parvum *were able to excrete oocysts after a second challenge with *C. bovis *but not with *C. parvum*. The authors concluded that immunity to *C. parvum *was not extended to *C. bovis*. Consistently, in our study, the presence in the same animal during sequential sampling of *C. parvum, C. bovis *and *C. ryanae *suggests that immunity against *C. parvum *and against *C. bovis *did not extend to *C. ryanae*. Furthermore, the observation that one animal excreted sequentially *C. parvum*, *C. ryanae *and *C. bovis *suggests that immunity against *C. ryanae *did not extend to *C. bovis *as well.

Finally, the risk to human health posed by *Cryptosporidium *infected cattle in France was assessed. The detection of *C. ubiquitum *(a rare infectious agent detected in humans [52]), *C. ryanae *and *C. bovis *(which are mainly specific for cattle) led to consider that the 22-week-old calves are not likely a public health concern. However, the major detection of *C. parvum*, a prevalent zoonotic species, in 5-week-old calves was in agreement with the report of Atwill et al., who considered that the contribution of cattle to human cryptosporidiosis is limited to calves under 2 months of age [53].

To determine *C. parvum *subtypes, the sequence analysis of a fragment of the gp60 gene was done. Our results show that in the region of Brittany, all identified *C. parvum *gp60 subtypes belonged to the IIa family which was previously found in both animals and humans [42]. Particularly, human infections with the IIa subtype are commonly seen in areas with intensive animal production [54]. Among the 48 gp60 subtypes formerly described in cattle [55], only six were identified in this work, being IIaA15G2R1 the most commonly found. This subtype has been widely reported in calves and humans in different countries such as in Portugal [54], Slovenia [56] and The Netherlands [57]. This observation confirms previous works and suggests a zoonotic transmission of the parasite also in this region.

It is noteworthy that the three predominant subtypes (IIaA15G2R1, IIaA17G1R1, and IIaA16G3R1) found in this work were also described in cattle with an equivalent distribution in The Netherlands [57] and England [40]. Thus, the subtype IIaA15G2R1 was found in 74.5% of the samples in this work, 68.9% in The Netherlands and 68.6% in England. The IIaA17G1R1 was identified in 11.7% of the samples in this report, 10.8% in The Netherlands and 13.8% in England. The IIaA16G3R1 determined in 5.9% of our samples, was characterized in 4.65% in The Netherlands and 5.8% in England. It is remarkable that subtypes, IIaA16G2R1, IIaA16G1R1 and IIaA13G2R1 were equivalently underrepresented in these three countries. This observation could suggest that the proportion of a gp60 subtype would not be randomly represented in a population.

Finally, the zoonotic transmission assessment of *C*. *parvum *in France would require a comparative investigation of variable genetic loci both in human and animal samples.

This is the first report on the molecular identification of *Cryptosporidium *species or genotypes in veal calves in France. According to data reported previously in many countries, a sequential distribution of species is observed in cattle according to age. *C. parvum *was mainly observed in the youngest calves, while *C. ryanae *and *C. bovis *became predominant in stool specimens collected in older animals. In some cases, several *Cryptosporidium *species were successively detected in the same calf, suggesting that the immune defense against *C. parvum *is not efficient against *C. ryanae *or *C. bovis*. Finally, the major identification of the IIaA15G2R1 subtype in France suggests that 5-week old calves could be a reservoir for zoonotic parasites transmissible to humans.

## Competing interests

The authors declare that they have no competing interests.

## Authors' contributions

JF and KG participated in the conception and design of the study, carried out the experiments and drafted the manuscript. HL designed the sampling strategy and collected samples on farms. JF, KG and AFD designed the protocol for molecular assay and participated in the analysis result. OHG carried out molecular assays. EDC, GC and PH participated in the coordination of the study and helped draft the manuscript. All authors read and approved the final manuscript.
